# Phenomenology’s place in the philosophy of medicine

**DOI:** 10.1007/s11017-023-09619-1

**Published:** 2023-03-24

**Authors:** Matthew Burch

**Affiliations:** University of Essex in the School of Philosophy and Art History and the Director of their Interdisciplinary Studies Centre, Colchester, UK

**Keywords:** Phenomenology of medicine, Health, Disease, Illness, Applied phenomenology

## Abstract

With its rise in popularity, work in the phenomenology of medicine has also attracted its fair share of criticism. One such criticism maintains that, since the phenomenology of medicine does nothing but describe the experience of illness, it offers nothing one cannot obtain more easily by deploying simpler qualitative research methods. Fredrik Svenaeus has pushed back against this charge, insisting that the phenomenology of medicine not only describes but also *defines* illness. Although I agree with Svenaeus’s claim that the phenomenology of medicine does more than merely describe what it is like to be ill, once one acknowledges its more far-reaching theoretical aspirations, one sees that it faces an even more difficult set of objections. Taking a cue from recent work by Rebecca Kukla, Russell Powell, and Eric Scarffe, I argue that the phenomenology of medicine could answer these objections by developing an institutional definition of illness. This not only allows the phenomenology of medicine to answer its critics, but it does so in a way that preserves its major achievements and extends its reach within the philosophy of medicine.

## Introduction

Once a minor movement, today the phenomenology of medicine (henceforth PM) exercises enough influence that critics feel the need to ‘put it in its place.’ Jonathan Sholl, for example, argues that PM’s proper place resides several notches below its current rank [1]. As a discipline that does nothing but describe the experience of illness, Sholl argues, it is unclear whether PM offers anything one cannot obtain more easily by deploying simpler qualitative research methods. Sholl is not alone in this view. For a growing chorus of critics, phenomenological work in the philosophy of medicine and adjacent fields calls to mind the fabled emperor, strutting through town, naked and shameless, boasting of virtues no one else can see [2–4].

In a recent paper, Fredrik Svenaeus dismisses these worries, arguing that critics like Sholl fail to appreciate PM’s distinctive offer [5]. PM does not merely describe experience; it also provides first-person definitions of key medical concepts such as health and illness. It cannot be replaced by other qualitative research methods, then, because those methods yield no such definitions.

In what follows, I weigh in on this conversation about the nature and value of PM. The paper has five sections. Section one defends Svenaeus’s claim that PM’s distinctive offer lies not merely in describing what it is like to be ill but rather in identifying the constitutive features of illness experiences. In section two, however, I argue that once PM’s aims are properly understood, it becomes clear that it faces a much more difficult set of objections; and in section three, I argue that these objections ultimately stem from a fundamental flaw in PM’s practice. Namely, it presupposes a normative notion of illness that its analyses cannot in principle provide. To overcome these worries, section four takes a cue from recent work by Rebecca Kukla and Russell Powell & Eric Scarffe and articulates an institutional account of illness [6–8]; and section five explains how this institutional approach would not only allow PM to answer its critics and preserve its major achievements, but it would also extend its reach within the philosophy of medicine.^1^

## What PM claims to do

According to critics like Sholl, if PM does nothing but describe experience, then it offers nothing distinctive, because countless research methods describe experience [1]. Moreover, some of those methods afford sophisticated tools and techniques to sort, systematize, and synthesize large amounts of first-person data, without subjecting readers to highfalutin disquisitions on ‘being-in-the-world.’ Why bother with phenomenology, then, when one can get the same results by less esoteric, more analytically powerful means?

The impression that PM does nothing but describe experience stems from multiple sources: (1) analytic philosophy and cognitive science have long used the term phenomenology as a synonym for ‘phenomenality,’ (2) enormous amounts of phenomenology-inspired qualitative research in fact does nothing but describe ‘lived experience,’ and (3) PM’s authors often insist that their key contributions lie in first-person descriptions of the experience of health and illness. So, it is easy to see why some critics think PM does nothing but describe experience.

But those critics are mistaken. PM’s major figures clearly present what they do as a kind of conceptual work that does not merely describe what it is like to be ill but also attempts to identify the constitutive features of the experience of illness and to thereby define illness (along with other key medical concepts).

How do PM’s authors do this conceptual work? The notion that phenomenology can clarify the essential or “eidetic” characteristics of different kinds of phenomena traces back to Edmund Husserl’s notion of the ‘eidetic reduction.’ To perform this reduction, the phenomenologist suspends her theories and factual beliefs about the phenomenon in question and examines the various ways it can be given; and based on that examination, she draws conclusions about possible conceivable phenomena and their essences. An important part of this process is what Husserl called ‘eidetic variation,’ wherein one imagines which changes the phenomenon in question can undergo while remaining the kind of phenomenon it is. In so doing, one isolates the parameters governing what it means to be the kind of thing in question. Hence, as Amy Thomasson explains, this process is key to understanding how phenomenologists practice ontology. Unlike today’s prevalent neo-Quinean conception of ontology as the study of “what does (and does not) ‘really’ exist” [9, p. 290], in the phenomenological tradition, ontology denotes the study of “both concepts/meanings and the essences they represent” [9, p. 290], i.e., ontology involves “an *a priori* analysis of the *possible* kinds, categories, or modes of being, and what their relations are” [9, p. 292, *original emphasis*]. Phenomenological ontology does not ask which things *exist (or are)*, but rather what it *means to be* different kinds of things. To paraphrase Heidegger, it is concerned not with *beings* but rather with the *meaning* of beings. By isolating the essential features of this or that phenomenon, one thereby specifies the criteria something would have to meet to count *as* an instance of that phenomenon.

These traditional phenomenological tools play an important role in PM’s approach to illness. To see how, one can look at the work of three major figures in PM, beginning with S. Kay Toombs. In a seminal paper, Toombs claims that her “phenomenological description of illness-as-lived reveals certain essential features that characterize this way of being and that pertain to the phenomenon of illness, *per se…*” [10, pp. 228–29]. In Husserlian terms, she aims to identify the “‘eidetic’ characteristics of illness,” i.e., those characteristics of illness that “remain unchanged regardless of any varying empirical features” [10, p. 229]. Toombs drives the idea home with an analogy: just as “the eidetic characteristics of a cube would include rectangularity, limitation to six squares, and corporeality” [10, p. 229] – a fact grasped through eidetic variation – she claims that the “eidetic characteristics of illness transcend the peculiarities and particularities of different disease states and constitute the meaning of illness-as-lived” [10, p. 229]. Among the eidetic characteristics of illness, Toombs identifies “the perception of loss of wholeness, loss of certainty, loss of control, loss of freedom to act, and loss of the familiar world” [10, p. 229]. This, in brief, is her eidetic – and so, in phenomenological terms, ontological – account of illness, since it defines the necessary conditions for something to count as – to *be* – an incidence of illness.

Svenaeus promises something similar. Responding to Sholl’s criticism, he claims that his work does not merely describe what it is like to be healthy or ill but also defines health and illness via purely phenomenological “constitutive analysis” [5, p. 468]. Although a phenomenologist consults his own experience and reads about illness experiences in “books, articles, blogs, etc.,” he defines health and illness by relying on the kind of eidetic variation described above, i.e., by “examining the way human experiences characteristically differ in situations of health and illness” [5, p. 467]. And so, Svenaeus claims, his ontological definition reveals what is “*constitutive* of illness” *per se*, i.e., it “show[s] us of what illness (and health indirectly) *consists*” [5, p. 468, *original emphasis*]. The definition Svenaeus arrives at via this method characterizes health as “homelike being-in-the-world,” a term of art designed to capture “the character of the normal, unapparent transparency of everyday activities” [11, pp. 233–4]. ‘Homelikeness’ is not about feeling *gemütlich* but rather a matter of being fully engaged in one’s projects. On the other hand, illness, according to Svenaeus, disrupts this ‘homelike’ existence, and so he defines it as “an unhomelike being-in-the-world in which the embodied ways of being-in of the person have been thwarted” [11, p. 233]. Thus, Svenaeus emphasizes the bodily nature of the disturbance, claiming that the ill body “shows up as an alien being (being me, yet not me) and this obstruction attunes the entire being-in-the-world of the ill person in an unhomelike way” [11, p. 233]; but he also highlights the affective dimension of illness, claiming that to be ill is “to find oneself in a pattern of disorientation, resistance, helplessness, and perhaps even despair” [11, p. 232; all passages from 11 here are also cited in 5, p. 463].^2^

Havi Carel also seeks to uncover the constitutive features of illness experiences, though it is not always clear whether she means to offer a phenomenology of serious somatic illness only, or, more ambitiously, a phenomenology of illness as such. In other words, there are two plausible ways to interpret Carel’s project. On a narrow interpretation, Carel offers a phenomenological account of a certain subset of serious, somatic, life-altering illnesses that share some core experiential features; but she does not define illness as such because she does not account for minor illnesses or mental illnesses.^3^ However, Carel’s text strongly supports an interpretation on which she offers a phenomenological account of illness per se and rejects the notion that mild health conditions count as illnesses at all. This latter reading is faithful to elements of Carel’s text, e.g., she presents her project as an attempt to identify which “characteristics unite all and only illness experiences” [12, p. 3] and which “changes in the global structure of experience…apply to many, or even all, illnesses” [12, p. 2]. Indeed, the book is called *Phenomenology of Illness*, which suggests an ambitious attempt to define not just a subset of illness experiences but rather illness as such. Although Carel’s text supports both interpretations, only the second reading is of interest here, because it resembles the approach Toombs and Svenaeus take – namely, it seeks to identify the constitutive features of illness as such, not simply a subset thereof – and so faces similar difficulties.

On this interpretation, Carel offers a purely phenomenological analysis of the life-altering conditions that properly count as illnesses. Ultimately, Carel’s analysis portrays illness as an “existential transformation” [12, p. 14] – “a complete transformation of one’s life,” one that radically disrupts and alters “one’s being-in-the-world, including one’s relationship to the environment, social and temporal structures, and one’s identity” [12, p. 37]. Illness disrupts one’s “habits, expectations, and abilities,” and this upheaval in turn throws off – and in extreme cases destroys – “the overall coherence of one’s life” [12, p. 14]. Carel particularly focuses on the way illness disrupts the ill person’s “sense of embodied normalcy” [12, p. 15]. “In a normal situation” [12, p. 30], she argues, “the healthy body” is “*transparent*: we do not experience it explicitly” [12, p. 55]; instead, it works quietly in the background, making it possible for one to focus on whatever project currently holds one’s attention. For Carel, this transparency of the body is “the hallmark of health and normal function” [12, p. 56] that gets disrupted in illness as the obtrusive “ill body thwarts plans, impedes choices, and renders actions impossible” [12, p. 42]. Again, on this reading, what Carel offers is not just a characterization of a subset of illnesses sufficiently similar to share some core experiential features, but rather a phenomenological analysis of illness per se.

These brief reconstructions suffice to show that critics are wrong to peg PM as merely describing what it is like to be ill. Among other things, it endeavors to develop eidetic/ontological accounts of key medical concepts like health and illness. When one sees that PM understands itself in these terms, however, it becomes clear that it faces a deeper set of problems.

## Three objections

In this section, I raise three objections to demonstrate that these authors fail to offer feasible eidetic/ontological accounts of illness. In the next section, I show that this failure flows from a more fundamental flaw in their approaches, and in section four I recommend a way to fix that flaw and thereby answer these objections.

The first objection targets the interpretation of Carel’s work according to which she attempts to define illness as such. On this reading, Carel maintains that experiences of non-life-altering conditions – e.g., colds, flus, sore throats, transient nausea, non-life-altering cases of coronavirus, and so on – do not count as illness experiences. This is a radically revisionary claim. Most people around the world consider minor conditions illnesses because they experience them as such; they feel “off” and take measures – including treatments and medicines – to mitigate their effects. Why should one think them wrong? It would be one thing if Carel offered a debunking argument that somehow justified such revisionism, but she does not.

Instead, on this interpretation, Carel excludes these experiences from the category of illness *before* she launches her investigation. She then analyzes experiences of serious, life-altering conditions to arrive at a phenomenological account of illness as an ‘existential transformation.’ But how can she exclude experiences of minor conditions from the category of illness prior to an analysis meant to furnish an account of illness that “unites *all* and only illness experiences” [12, p. 3 *emphasis added*]? It would be one thing if she began her analysis with everything considered an illness experience and then refined things from there, ruling minor conditions out on some principled basis. But she does not. On this interpretation, before her analysis begins, she excludes experiences that most people consider illness experiences from the category of illness. This implies that she already has access to the norm that her analysis aims to furnish. Not only is such a view radically and gratuitously revisionary, but it presupposes access to the very norm it promises to provide.

Shifting focus now to Toombs and Svenaeus, in the phenomenological tradition, an eidetic/ontological account of some phenomenon purports to identify its defining marks or constitutive features. Such an account, then, should cover all phenomena of the relevant kind. Think of Toombs’s analogy to the eidetic analysis of a cube. On that analysis, no figure that lacks rectangularity, six square sides, or corporeality can count as a cube. Eidetic accounts, then, are supposed to capture every single instance of the target phenomenon. Thus, on the approach taken by Toombs and Svenaeus, if one can identify *one* bona fide illness experience that lacks *one* aspect of their putatively eidetic/ontological accounts, then those accounts fail and stand in need of revision.

To find such a counterexample, one can turn once more to minor illnesses. A minor illness does not involve the dramatic losses that Toombs identifies as eidetic characteristics of illness. Unless one’s health is already compromised by another condition, when one catches a mild cold, one does not lose one’s sense of wholeness, certainty, control, or freedom, and the world does not become alien or unfamiliar. Neither does Svenaeus’s definition fit the experience; again, unless one’s health is compromised by another condition, a mild cold does not transform one’s body into an alien being, making one not-at-home-in-the-world, nor does one find oneself in “a pattern of disorientation, resistance, helplessness,…[or] despair.” Toombs and Svenaeus successfully capture important features of some illness experiences, but as eidetic/ontological accounts, they fail, because they cannot capture minor illnesses like colds.

I want to now briefly address three things. First, I am not just being a pernickety stickler. By claiming to identify the “eidetic characteristics” [10, p. 229] that are “constitutive of illness” as such [5, p. 468], Toombs and Svenaeus are the ones who set the bar so high that a single counterexample defeats their claims. Secondly, this counterexample is far from trivial: minor illnesses account for a vast swathe of illness experiences that these accounts fail to capture. Finally, Toombs could retreat to a position like Carel’s which simply excludes minor conditions from consideration as illnesses, but, as shown above, that is not a particularly attractive option. Svenaeus does not have this option at all, because he explicitly claims that his definition encompasses minor illnesses like colds [5, p. 470].

Turning now to my third and final objection, I want to raise a worry about PM’s normalcy talk. As shown above, Carel claims that in “a normal situation” [12, p. 30], “the healthy body” is “*transparent*” [12, p. 55, *original emphasis*]; and that such transparency is “the hallmark of health and normal function” [12, p. 56]. Similarly, Svenaeus uses ‘homelikeness’ – his term for health – to capture “the character of the normal, unapparent transparency of everyday activities” [11, p. 234]. In the paper discussed above, Toombs works with a largely implicit commitment to a standard of normal embodiment, i.e., a norm against which she describes the various losses she links to illness [10]; however, in another influential paper published a year later, Toombs explicitly puts normalcy at center stage, consistently contrasting illness experiences to life’s “normal course of events” [13, p. 211].

This normalcy talk is objectionable because it illicitly exploits an ambiguity in the word ‘normal.’ Normal can mean statistically typical (as in, it is normal to have ten fingers), and it can also take on a normative significance when paired with the term “pathological” in a conceptual binary (as in, paedophilia is not normal but rather pathological). PM’s authors talk about the body’s transparency as if it is “normal” in both the statistical and normative sense. Strictly speaking, both claims are incorrect. In the phenomenological tradition, the transparency of the ‘lived body’ – the body as it is experienced first-personally – is a condition for the possibility of intelligible experience. The lived body’s transparency, then, is not simply statistically typical – like having ten fingers – but is rather a constitutive feature of intelligible experience as such. Moreover, this holds true of the lived body both in a healthy “normal” condition and an ill “pathological” one. Even in serious, life-altering illness, when part of the body becomes obtrusive, obstinate, or conspicuous, the lived body, for the most part, remains transparent and continues to play its constitutive role.

For example, say one morning I wake to find my left leg partially paralyzed. That leg will become urgently conspicuous, which will indeed highlight ways in which it had been experientially transparent. However, when I set out to investigate the extent of the damage, my postural control, the bodily skills of my torso, arms, and hands, the saccadic movement of my eyes, my mastery of the associated sensorimotor contingencies, and so on, will *remain experientially transparent*, making it possible for me to focus on and explore the conspicuous limb. To experience my illness as an illness, my body must remain largely transparent. From a phenomenological standpoint, then, it is false to claim that the “normal,” healthy body is transparent and the ill body conspicuous; the lived body is always partially transparent in intelligible experience, whether healthy or ill. Furthermore, the transition from transparency to obstinacy is in no way distinctive of illness but rather a core feature of embodiment evident in experiences that involve no illness whatsoever, e.g., when I sit too long at a conference, and my leg goes numb, reminding me to get up and move.

In sum, then, these authors’ analyses of the lived body do not justify but rather presuppose the legitimacy of their normalcy talk within the framework of PM.^4^ So, when these accounts contrast illness with the normal course of events, they do so not with a conceptually clarified notion of normalcy, but rather with an ambiguous sense of normalcy that they take for granted. This is a serious problem, because the notion of illness depends fundamentally on a distinction between normal and pathological suffering. When one experiences painful muscle soreness the day after an ill-advised workout too difficult for one’s current fitness level, one is not ill, but merely sore; when one experiences the exact same sort of soreness due to a virus, one is not merely sore, but ill. In the relevant sense of the word “normal,” soreness after the workout is normal, but soreness due to the virus is not. The analyses of the lived body discussed above fail to furnish a norm that makes that distinction possible. Instead, their references to the healthy body as “normal” presuppose such a norm.

## A more fundamental flaw

As I said at the beginning of the previous section, the three objections just considered arise from a more fundamental flaw in PM’s practice. To bring this flaw into view, I turn to recent work by Kukla and Powell & Scarffe [6–8]. These authors identify a flaw in today’s three dominant approaches to defining disease; and I argue that the phenomenological definitions of illness discussed above suffer from the same basic flaw.

Before I do so, however, I want to make something explicit to avoid unnecessary confusion: to my knowledge, the research programs undertaken by Kukla and Powell & Scarffe are entirely independent of each other. Kukla’s piece was published in 2015 [6], the two relevant pieces co-authored by Powell & Scarffe were published in 2019 [7, 8], and the latter do not cite or mention the former. Here, then, are two independent research programs that happen to develop very similar critiques of the contemporary philosophical discourse on disease. I do not mean to equate their views, as they differ in several important respects.^5^ For my present purposes, however, it will be important to focus on what they have in common.

To begin, here is a brief description of the dominant approaches to disease that these authors criticize:

1. Disease normativism maintains that the notion of disease is a social construction that reflects nothing more than value judgements about biomedical states [14].
2. Disease naturalism attempts to define disease in value-neutral terms as biological dysfunction: a normally functioning biological trait makes a statistically normal contribution to an organism’s evolutionary fitness, and a trait that departs from normal function to a stipulated degree is deemed biodysfunctional or a disease [15].
3. Hybrid views combine elements of 1) and 2). The most influential version defines disease as ‘harmful dysfunction’ - the judgment that a biomedical state is ‘harmful’ incorporates the evaluative dimension of normativism, and the claim that the state is biodysfunctional incorporates the putatively value-neutral element of naturalism [16].

Kukla and Powell & Eric Scarffe bring the same basic charge against these views – none furnishes a sufficiently normative concept of disease for the institutions of medicine. To be clear, these authors are pluralists about disease concepts; they acknowledge that there may be multiple valid concepts of disease suitable for different projects. For example, they accept that a strictly scientific concept of disease might be suitable for biological research. They insist, however, that only a normative notion of disease can do the work one needs such a concept to do in the institutions of *medicine*, because medicine is a fundamentally “normative project” [6, p. 516]: the “institutions of medicine are designed, first and foremost, to promote, restore, and protect health”, and doing so is “an important component of justice” [6, p. 515]. Disease diagnosis plays an important role in that normative project as it explains that “some biomedical state is disvaluable because it significantly interferes with well-being, flourishing, or opportunity, which in turn has important ramifications for how limited health care resources are prioritized” [8, p. 1176]. In this way, the concept of disease does important *institutional* work to help make *normative* calls about health policy, treatment decisions, and the just distribution of resources. At the individual level, disease diagnosis signals that one is entitled to support; and at the population level, it serves as a heuristic to ensure that the relevant decision makers justly and equitably prioritize the treatment of those who, biomedically speaking, are worst off. The three approaches in disease discourse briefly described above cannot play this normative institutional role, because they are purely *descriptive*: disease naturalism describes biological facts about functions and fitness; disease normativism describes social facts about which biomedical states a community happens to disvalue; and hybrid views describe the conjunction of these social and biological facts [7, p. 582]. Thus, none of these views specify which biomedical states one *should* disvalue. As Kukla puts it, these views are “devoid of normative force or practical upshot” [6, p. 515]. In light of this mismatch between the normative requirements of the institutions of medicine and the purely descriptive character of today’s dominant definitions, Kukla and Powell & Scarffe offer new, substantively normative definitions to fill the gap.

From here on, I will focus mostly on Kukla’s approach, because I find it more congenial to my own aims and I think it better demonstrates the problems with the definitions of illness at work in the PM approaches described above. Kukla begins their analysis by defining health and health conditions; then they define the concept of disease in relation to the concept of a health condition. According to Kukla, health conditions cannot be captured in purely social constructionist or scientistic terms; rather, in determining what counts as a health condition, one is constrained both by social *and* natural facts [6, p. 526]. Health conditions can only take on their particular meaning in – and so are dependent on – the social institution of medicine; but the fact that they count as health conditions also depends essentially on the empirical conditions of the body in its environment. With this dual nature of health conditions in view, Kukla offers their *Institutional Definition of Health*:

A condition or state counts as a *health condition* if and only if, given our resources and situation, it *would be best for our collective well-being* if it were medicalized—that is, if health professionals and institutions played a substantial role in understanding, identifying, managing and/or mitigating it. In turn, *health* is a relative absence of health conditions (and concomitantly a relative lack of dependence upon the institutions of medicine) [6, p. 526].

Based on this account of health conditions, Kukla then offers the following institutional definition of disease: “Roughly, we can think of a disease as a repeatable, relatively stable bodily state or process that systematically causally contributes to one or more health condition” [6, p. 527]. Kukla’s definition, then, is intrinsically normative – it defines health conditions in terms of *what would be best* for “our collective well-being” [6, p. 526]. Identifying something as a health condition is inseparable from the normative judgment that one *ought* to medicalize it – that the condition is *properly* considered a health condition because it tends to interfere with human flourishing, and it would be a good thing to bring the tools of medicine to bear on it. With this normative dimension, Kukla’s definition can do the ethical work one needs it to do in the practice of medicine.

It is important to emphasize the realist implications of an institutional approach: it implies that one can *discover* that a condition is properly considered a health condition by *learning* that it would be helpful to medicalize it; conversely, one can also discover that one has *mistakenly* medicalized conditions by learning that it is not helpful to bring the practice of medicine to bear on them. As an example of the latter, consider the case of homosexuality. Until recently, the American Psychiatric Association, the WHO, and a good deal of the wider public considered homosexuality a disease. The normative concepts of health and disease developed by Kukla and Powell & Scarffe allow one to state unequivocally that this call was terribly, horribly wrong. One learned that medicalizing and “disvaluing homosexuality is not rationally justified” because doing so “causes objective harm and injustice” [7, p. 582]. Homosexuality, under this analysis, was never a disease.

This, however, does not imply that what one learns in such cases are facts that exist apart from social practices; rather, whether one should medicalize some condition depends on “our resources and situation” [6, p. 525], which entail “all sorts of changeable and human-practice-dependent facts, including what medical techniques and interventions are available and the cultural context in which they will be used, among other factors” [6, p. 527]. Thus, the validity of normative judgments about medicalization depends crucially on the actual institutional context. Dyslexia, for instance, might be properly disvalued and so appropriately medicalized in an institutional setting where literacy tends to shape a person’s life prospects, while it would be permissible to ignore dyslexia in a culture where no one reads. Thus, Kukla and Powell & Scarffe argue that when one asks whether one should medicalize a condition, one cannot simply focus on the body as if it exists in an “institutional vacuum” [7, p. 583]; one must also, to the best of one’s ability, bring the social-material environment into view and try to determine whether one is looking at a health condition in need of medical attention, an institution in need of reform, or some combination [6].

Sensitivity to the institutional setting, however, does not make judgments about which conditions one should medicalize any less normatively substantive. Such judgments represent all-things-considered views about the way the world is and what is best to think and do.^6^ Judgments about health conditions are objective in the sense that they are grounded in empirical knowledge about the body in its environment, the “extension [of which] is not simply up to us” [6, p. 527] and they are normatively binding, identifying the conditions that one *should* medicalize.^7^

With this institutional definition of disease in hand, I can now fulfil the promise I made at the start of this section, namely, to shed light on the more fundamental flaw that underlies the objections from the last section. To briefly recall those objections: (1) on the interpretation according to which Carel offers a phenomenology of illness as such, she presupposes the concept of illness her analysis aims to provide; (2) Toombs and Svenaeus each provide a putatively eidetic/ontological account of illness that fails to capture an enormous swathe of illness experiences; and (3) PM’s normalcy talk presupposes access to a distinction between the “normal” healthy body and its pathological state. The institutional definitions just discussed, I contend, show why these problems are inevitable.

Illness, like disease, is a normative institutional concept. What counts as an illness experience depends on normative judgments regarding which experiences *ought to* count as illness experiences.^8^ But instead of making a normative case for which experiences one *should* count as illness experiences, PM’s authors simply identify the common characteristics of experiences that they already consider illness experiences. Thus, their approach *necessarily* presupposes a normative understanding of illness – which their non-normative analyses of illness experiences cannot in principle provide.

This explains why the problems identified in objections (1) and (3) are inevitable – these authors take for granted access to concepts of illness and the “normal” body that they cannot explain nor justify, because illness and normality are *normative* concepts that one cannot construct merely by identifying the constitutive features certain experiences share in common. Such concepts, rather, are already at work when these authors select the experiences they want to analyze. Some version of objection (2) is also inevitable – there will invariably be counterexamples to any purely phenomenological description of the common features of illness experiences, because one cannot determine what counts as an illness merely by describing experiential commonalities. What counts as an illness hangs on what one thinks is best to treat as an illness, which depends on one’s resources, situation, and a lot of practice-dependent contingent facts; and one has no reason to expect that these things will, by some miracle, strictly covary with a uniform set of experiential characteristics. For instance, one might think it best, all things considered, to treat the experience of food poisoning, major depression, and the stages of suffering that arise from emphysema as illness experiences. There is no reason to shoehorn such radically disparate experiences into some vague, putatively all-encompassing phenomenological “definition,” because, again, one needs to consider much more than experiential similarities when deciding which experiences should be classified as illness experiences. Try to force all illness experiences into a common phenomenological frame and one will inevitably face counterexamples, unless you fall back on a phenomenological “definition” so vague that it captures veritably any experience of discomfort. In short, one should not expect to find a core set of phenomenological features that all illness experiences share, because illness is not a descriptive but rather a normative concept designed to do important work in the institutions of medicine.

There is something worth noting at this point: If this argument is correct, then PM has no real cause for embarrassment over its fundamental flaw. Why? Because the argument suggests that the flaw in question features in a wide range of positions in the philosophy of medicine, not just phenomenological ones. What Kukla and Powell & Scarffe argue, after all, is that most attempts to define disease overlook the need for a substantively normative definition. PM has essentially overlooked the same thing with respect to illness, and so has made a version of the same common mistake.

Now, some of PM’s authors might push back here, arguing that while the concept of disease might belong to the institutions of medicine, health and illness do not. For instance, Svenaeus argues that although illness *tends* to correlate with disease, one should “allow for the possibility of illness without disease, as everyday experience and not medical science has the final word in defining health or illness” [5, p. 464]. Similarly, although Carel seems to endorse an intrinsic link between disease and illness when she claims that “Illness is the experience of disease,” she also suggests that a person can be “ill but not diseased” [12, p. 17]. So, it seems, these authors might reject the institutional approach as the by-product of a bankrupt biomedical model of health that refuses to give everyday experience its due, when it is precisely everyday experience that matters in defining illness.

In this context, however, insisting on the conceptual independence of health and illness would be a mistake for at least three reasons. First, when Svenaeus and Carel make these claims, their target is a kind of biomedical science that considers everyday experience irrelevant to the project of defining health-related concepts. Institutional approaches, however, conceptualize health conditions as conditions that those who depend on the institutions of medicine to promote, restore, and protect their health *should* disvalue and medicalize in light of what is best for their collective wellbeing. Far from ignoring it, then, institutional approaches factor everyday experience into its deliberations about which bodily states one should disvalue and medicalize.

Secondly, an institutional approach does not conceive of medicine as existing apart from the lifeworld, like an alien observer objectifying human reality from the outside. Rather, it sees medicine as a practice within the lifeworld, organized around our shared interests in promoting, restoring, and protecting health. So, there is nothing intrinsically scientistic or “de-worlding” about thinking that what counts as an illness is partially determined by the social practice of medicine. That practice is part of the lifeworld.

Finally, allowing for the possibility of illness in the absence of any health condition has significant downsides. Consider the case of homosexuality again. In the recent past, when homosexuality was widely considered a disease, many homosexuals viewed their sexual orientation as an illness. Now, most hold that homosexuality never was a disease and that those who saw their sexual orientation as an illness were *wrong*. Hostage to an oppressive ideology, they *misinterpreted* their own experience. However, if a person can be ill in the absence of any health condition, then any gays and lesbians who internalized their oppression, felt alienated from their desires, and saw their experience as fitting Svenaeus’s description of ‘unhomelikeness,’ *ipso facto* suffered from an illness. And if meeting some phenomenological descriptions were all that mattered for determining whether an experience is an illness experience, then there would be no normative basis for respectfully suggesting that they were getting it wrong. In a word, such an approach would make the notion of illness absurdly relativistic. The stigmatized and self-loathing gay Texan teen who feels ‘unhomelike’ in his conservative Baptist milieu would suffer from an illness, while his ‘at-home-in-the-world’ California counterpart would be healthy. Indeed, any person struggling with life’s problems who sees herself in one of these phenomenological descriptions of illness could count herself ill. Thus, the suggestion that one ought to allow for illness in the absence of any health condition raises serious concerns about mistakenly medicalizing internalized oppression and pathologizing normal suffering. Let illness float free from its foundation in a health condition, and you forfeit the realist implications of the institutional approach.

## Towards an institutional definition of illness

One has good reason, then, to insist on an intrinsic link between the experience of illness and the presence of a health condition. But how should one conceptualize that link?

One might be tempted to adopt and adapt a division of labor familiar from PM: one could allow philosophers like Kukla and Powell & Scarffe to define health, while claiming illness for phenomenology. Thus, one might conceive of illness as the aversive, flourishing-undermining bodily experiences that arise from a health condition. This division of labor would allow phenomenologists to focus strictly on first-personal analyses of the bodily experiences arising from health conditions, while other philosophers did the work of defining health, health condition, and disease.

This version of PM would still count as an institutional account of illness because it would link illness intrinsically to the presence of some health condition, institutionally defined. So, for example, say two people experience comparable episodes of extremely low mood that could be described in experientially similar terms; however, one person has just suffered a bereavement and the other can point to no reason for her crushing feelings of despair. Experiential similarities notwithstanding, the phenomenologist who takes the institutional approach would only see the second case as an illness, because the institutions of medicine, appropriately, do not think it right or helpful to medicalize our initial depressive responses to bereavement. On this approach, then, illness is an institutional concept because what counts as an illness hangs on what counts as a health condition, institutionally defined.

To be clear, on the approach just sketched, not all health conditions would involve illness. For example, one might consider shortness due to human growth hormone deficiency a health condition, but it would not involve illness, because any aversive bodily experiences associated with shortness are due to the social context and human cruelty, not shortness itself. Hence, the approach would also allow one to distinguish between illness and oppression, as illness would be understood as the aversive bodily experiences arising not from the stigmatization of a health condition but rather from the health condition itself.

The proposed approach, however, will not quite work, because not every flourishing-undermining, aversive bodily experience that arises from a health condition is properly considered an illness. Imagine another scenario: two people have roughly the same flourishing-undermining aversive bodily experiences arising from two different health conditions, namely, an infection and an injury. Even if their aversive bodily experiences border on identical – say, pain and swelling in the affected limb that prevents them from engaging in their ordinary activities – only the aversive bodily experiences arising from the infection would count as illness experiences. Why? Because it is considered best, from the standpoint of the practice of medicine, to treat aversive bodily experiences arising from injury differently than one treats those arising from infection. Like the institutional definition of a health condition, the institutional definition of illness will be constrained by our social practices and by the reality of the body in its environment. Injuries and infections are different material realities that call for different tools, techniques, and treatment regimes. So, from the standpoint of the aims of the practice of medicine, there are good reasons to place them in different categories. This means that the tempting option discussed in the previous three paragraphs will not suffice. That is, one cannot define illness as the aversive bodily experiences arising from a health condition, because not all such experiences count as illness experiences.

Illness, then, cannot simply piggyback on the institutional definition of a health condition; instead, it will need its own institutional definition. In other words, one also needs to think through what is best, given one’s resources and situation, to regard as illness experiences. On this approach, then, one would think of illness experiences - in a rough, preliminary way - as those flourishing-undermining, aversive bodily experiences that arise from a health condition and that it would be best for our collective well-being to medicalize. Of course, this raises the question – *which* subset of aversive bodily experiences would it be best to medicalize?

Answering this question in detail is beyond the purview of the current discussion, but I have already specified the relevant subset somewhat: the relevant aversive bodily experiences must arise not from the social oppression of those with a health condition but rather from the health condition itself. Moreover, the relevant aversive bodily experiences must not arise from injuries, because, for institutional reasons, those cases belong to a distinct treatment category. And there is no doubt that many other flourishing-undermining, aversive bodily experiences arise from a health condition that, for institutional reasons, should not be treated as illness experiences. For example, it may be common for patients living with a serious health condition to experience flourishing-undermining anxiety associated with their condition; but one might think it best, all things considered, not to medicalize that dimension of their suffering, because, like grief in bereavement, anxiety in the face of one’s mortal frailty strikes one as appropriate.

The larger point here is that working out precisely which subset of flourishing-undermining aversive bodily experiences one should count as illness experiences is not as straightforward as simply identifying which ones arise from a health condition. One must deliberate about which aversive bodily experiences it is best to consider illness experiences given the aims of the institutions of medicine and one’s resources, situation, and a host of practice-dependent contingent facts.

This implies that one should never consider one’s judgments about these matters final; rather, one should see them as at stake in the evolving institutions of medicine. After all, one can get these things wrong (i.e., one can wrongly medicalize bodily states and experiences that should never come under the scope of medicine). Furthermore, what counts as an illness will evolve as medicine develops and new treatment possibilities emerge. The point is not to pin down, once and for all, which bodily states count as health conditions or diseases. The point is rather to bring home the fact that making such determinations calls for ongoing philosophical and ethical deliberation. Thus, one should treat the questions of which bodily states should count as health conditions (or diseases), and which aversive bodily experiences should count as illness experiences, as in principle open to future deliberation. And one should never presume that one can know *a priori* which bodily states and aversive bodily experiences ought to be medicalized.

Now, given their common link to health conditions, the reader might want to know how the appropriate medicalization constitutive of illness experiences differs from that constitutive of disease. This issue is also too complex to explore fully here, but if one compares my rough institutional definition of illness to Kukla’s rough institutional definition of disease, one can highlight a significant difference in focus in these two definitional projects. I define illness experiences, roughly, as flourishing-undermining, aversive bodily experiences that arise from a health condition and that it would be best to medicalize; and Kukla defines disease, roughly, as “a repeatable, relatively stable bodily state or process that systematically causally contributes to one or more health condition” [6, p. 527]. This implies a significant difference in orientation when it comes to the task of identifying each. When it comes to disease, one will focus much more on the bodily states or processes and their putative causal contribution to one or more health conditions; and when it comes to illness, one will concentrate more on the flourishing-undermining aversive bodily experiences – or experiences of suffering – that arise from health conditions.

But one must be clear that this difference in overall orientation in no way recapitulates the division of labor I rejected above, namely, that division wherein philosophers deal with disease with an exclusively third-personal approach and phenomenologists tackle illness with their strictly first-personal method. As I argued in section three, everyday first-person experiences will factor into deliberations about what counts as a health condition, and so such considerations will shape the project of identifying diseases. What is more, as was shown in section three, a strictly first-personal approach to illness experiences is out of the question, because one cannot divorce the concept of illness from the notion of a health condition. So, in terms of overall orientation, the task of medicalizing disease will focus more on bodily states and processes and their causal relation to health conditions, while the task of medicalizing illness will focus more on aversive bodily experiences that arise from health conditions. But this overall orientation will not reflect a neat and tidy division of labor. Both projects will require interdisciplinary conversations in the institutions of medicine that span everything from the body’s material reality to fine-grained details about patients’ lived experiences.

## Phenomenology’s place

Shifting focus back to the big picture, what does this discussion reveal about phenomenology’s place in the philosophy of medicine? I have argued that PM cannot furnish definitions of illness by merely identifying the constitutive features of putative illness experiences, because such a procedure necessarily presupposes the very concept it promises to provide. In making this argument, have I not, in effect, answered Sholl’s call to put phenomenology in its place by banishing it to some irrelevant backwater in the philosophy-of-medicine empire?

To the contrary, the view I defend here in fact broadens the prevailing understanding of what phenomenology has to offer the philosophy of medicine. As has been shown, PM, by and large, has settled for a division of labor wherein biomedicine defines disease and phenomenology defines illness. But with an institutional approach, that division of labor collapses, and a normative, interdisciplinary approach becomes the default for defining *all* health-related concepts.

This creates at least two new openings for phenomenology to contribute to the philosophy of medicine. First of all, the institutional task of identifying which bodily states and aversive experiences one should medicalize requires normative resources to determine which states and experiences one ought to disvalue; and the phenomenological tradition boasts a wide variety of such resources.^9^ To take just one example, since one can frame the question of “collective wellbeing” and what one ought to disvalue in terms of human flourishing, one could follow the phenomenological approach Irene McMullin takes in *Existential Flourishing*. In that work, McMullin marshals phenomenological resources to argue that human beings always find themselves in the grips of claims from three normative domains – i.e., “the claims posed by the self, other, and shared world” [19, p. 68] – and that human flourishing consists in simultaneously responding well to all three sets of claims. Such a phenomenological account of human flourishing could serve as a normative foundation for reflecting on which biomedical states one ought to disvalue. One could argue that a health condition is a bodily state that (a) tends to interfere significantly with one’s capacity to flourish as construed on McMullin’s phenomenological account and (b) it would be helpful to medicalize. But McMullin’s view is just one option from a menu of phenomenological accounts that could potentially play this role. Secondly, although phenomenology cannot decide on its own which experiences ought to count as illness experiences, it can still provide phenomenological analyses of different aversive bodily experiences that are candidate illness experiences, and these analyses could feed important insights into the all-things-considered judgment about which experiences *should* count as illness experiences. Thus, when it comes to the institutional project of defining health-related concepts, phenomenologists need not sit on the sidelines, because they have a range of relevant resources *par excellence *to bring to bear on that task.^10^

Moreover, PM can also carry on doing what it has done all along, namely, carefully describing the constitutive features of subjectivity at play in illness experiences. Now, however, they can do so without worrying about the objections raised in section two, nor the fundamental flaw highlighted in section three, because they no longer need to take a normative conception of illness for granted. Rather, they can restrict their analyses to aversive bodily experiences identified as proper illness experiences within a normative institutional framework.^11^

## Conclusion

Important work in PM is vulnerable to various objections because it suffers from a fundamental flaw: it presupposes a normative notion of illness that its analyses cannot in principle provide. This flaw, however, is by no means fatal. Instead of taking for granted a normative distinction between the normal and the pathological and limiting itself strictly to the task of describing illness experiences, PM could contribute to the normative institutional project of defining health-related concepts, including illness. Furthermore, with that normative conception of illness in hand, PM could continue its work with more confidence that its analyses target experiences properly considered illness experiences. In sum, this move would allow PM to preserve its most significant achievements, answer its critics, fix its fundamental flaw, and extend its reach in the philosophy of medicine.

## Footnotes

1 I want to thank two anonymous referees whose comments improved this article.

2 One should note how odd it is for the phrase “and perhaps even despair” to appear in a definition — defining features should not be optional.

3 Some aspects of Carel’s text support this reading. First of all, at various points, Carel distances herself from the kind of *a priori* eidetic/ontological claims that Toombs and Svenaeus make. To take one example, after summarizing Toombs’s eidetic analysis of illness, Carel claims that in her work the common features of illness “should be understood in a more restrictive sense as not entirely eidetic, but as offering a general characterization of the experience of illness as lived by conscious adults with a certain degree of self-awareness, in Western societies” [12, p. 45]. Another passage that supports this narrow interpretation is a footnote in which she reminds the reader that she “use[s] the term illness to denote serious, chronic, or life-threatening illness, rather than common, transient illness such as the common cold” [12, p. 87, note 2]. Here, she explicitly refers to the common cold as an *illness*, which implies that she does consider it an illness, just not one that belongs to the subset of illnesses that interests her. Although Carel leaves the methodological remarks cited here fairly undeveloped in *Phenomenology of Illness*, I think she could profitably develop them in line with the approach I defend below in section four.

4 Sholl also points out that phenomenological discourse takes a notion of normality for granted, but he does not criticize the use of the term normal on phenomenological grounds [1, pp. 400–403].

5 I explore some of these differences below in footnote 7.

6 Although many disease theorists will blanch at this objective notion of normativity, as Powell & Scarffe point out [7, 8], the majority of bioethicists work under the assumption that one can objectively justify our judgments about biomedical states without appealing to a strong strand of moral realism [17].

7 Although these matters are complex and I cannot do them full justice here, I wanted to briefly explain my preference for Kukla’s approach. Powell & Scarffe offer the following new hybrid definition of disease: “a biomedical state is a disease only if it implicates a biological dysfunction that is, or would be, properly disvalued” [7, p. 582]. Their definition thus includes two objective components: “biological dysfunction and rational moral justification” [7, p. 582]. Although I find this definition compelling in many respects, I prefer Kukla’s for five reasons: (1) as Powell & Scarffe point out, including biological dysfunction as a component of their definition means it is unclear whether they can account for diseases associated with senescence, because these seem to result not from an evolutionarily defined biodysfunction but rather from “*the lack of selective investment* in tissue maintenance and repair in postreproductive phases of the lifespan” [7, p. 585, *original emphasis*]; (2) Powell & Scarffe argue that dispensing with the biological dysfunction component would make the disease concept unable to “prioritise biomedical conditions that tend to interfere most with well-being, flourishing and opportunity” [7, p. 586], but this seems wrong to me — it seems one could keep track of which biomedical conditions most interfere with flourishing without appealing to biodysfunction; (3) Kukla’s approach seems more promising for thinking about mental health conditions — one may well want to medicalize some mental health conditions without being able to pin down any associated biodysfunctions; (4) as Kukla points out, one cannot always trace a health condition “to the malfunctioning of a single part or subsystem of the body”, e.g., consider “depression, morbid obesity, and malnutrition” [5, p. 516]; (5) finally, as I explore in what remains of this section, Kukla provides a useful framework for thinking about illness.

8 How the institutional task of deciding what counts as an illness differs from the institutional task of deciding what counts as a disease is something I address, albeit in a preliminary way, at the end of this section.

9 For an overview and assessment of these resources, see Drummond [18].

10 This work, I contend, should be conceived of as a kind of applied phenomenology, because it uses the tools of phenomenology in conjunction with resources from other disciplines to tackle problems that lie beyond the purview of purely phenomenological inquiry. For a discussion of this conception of applied phenomenology and how it compares to other such conceptions, see Burch [20].

11 Of course, if phenomenologists want to avoid the normative work of developing institutional definitions of health-related concepts, they can simply continue to restrict their work to phenomenological analyses of (a) illness experiences identified as such by other philosophers or (b) “candidate illness experiences” not yet identified as proper illness experiences. In the latter case, they would simply need to withhold judgment about whether the target experiences properly count as illness experiences.

## References

[1] Sholl Jonathan (2015). Putting phenomenology in its place: some limits of a phenomenology of medicine. Theoretical Medicine and Bioethics.

[2] Ferry-Danini Juliette (2019). Should phenomenological approaches to illness be wary of naturalism? *Studies in history and philosophy of Science Part C: studies*. History and Philosophy of Biological and Biomedical Sciences.

[3] Gergel Tania L (2012). Medicine and the individual: is phenomenology the answer?. Journal of Evaluation in Clinical Practice.

[4] Paley John (2017). Phenomenology as qualitative research: a critical analysis of meaning attribution.

[5] Svenaeus Fredrik (2019). A defense of the phenomenological account of health and illness. The Journal of Medicine and Philosophy: A Forum for Bioethics and Philosophy of Medicine.

[6] Kukla Rebecca, Arras John D, Fenton Elizabeth, Kukla Rebecca (2015). Medicalization, normal function, and the definition of health. The routledge companion to bioethics.

[7] Powell Russell (2019). Rethinking “disease”: a fresh diagnosis and a new philosophical treatment. Journal of Medical Ethics.

[8] Powell Russell (2019). Rehabilitating “disease”: function, value, and objectivity in medicine. Philosophy of Science.

[9] Thomasson Amie L (2019). What can phenomenology bring to ontology?. Res Philosophica.

[10] Toombs S, Kay (1987). The meaning of illness: a phenomenological approach to the patient–physician relationship. Journal of Medicine and Philosophy.

[11] Svenaeus, Fredrik. 2013. Naturalistic and phenomenological theories of health: Distinctions and connections. *Phenomenology and naturalism: Exploring the relationship between human experience and nature*, eds. Havi Carel and Darian Meacham, 221–38. Cambridge: Cambridge University Press.

[12] Carel Havi (2016). Phenomenology of illness.

[13] Toombs S, Kay (1988). Illness and the paradigm of lived body. Theoretical Medicine.

[14] Engelhardt, Tristam H. 1995. *The foundations of bioethics*. Second Edition. New York: Oxford University Press.

[15] Boorse Christopher (1977). Health as a theoretical concept. Philosophy of Science.

[16] Wakefield Jerome C (2007). The concept of mental disorder: diagnostic implications of the harmful dysfunction analysis. World Psychiatry.

[17] Roache Rebecca (2018). Psychological disadvantage and a welfarist approach to psychiatry. Philosophy Psychiatry & Psychology.

[18] Drummond John J (2021). Phenomenological method and contemporary ethics. Continental Philosophy Review.

[19] McMullin Irene (2019). Existential flourishing: a phenomenology of the virtues.

[20] Burch, Matthew. 2021. Make applied phenomenology what it needs to be: an interdisciplinary research program. *Continental Philosophy Review *54(2): 275-293.

